# Primary hyperparathyroidism in a cat with synchronous bilateral parathyroid carcinomas: a case report

**DOI:** 10.3389/fvets.2026.1930590

**Published:** 2026-10-29

**Authors:** Ulsoo Choi, Guhyun Kwon, Jae-hyun Lim, Sang-Ik Oh, Sunjae Ock

**Affiliations:** 1 Department of Veterinary Clinical Pathology, College of Veterinary Medicine, Jeonbuk National University, Iksan, Republic of Korea; 2 Daegu Animal Medical Center, Daegu, Republic of Korea; 3 Department of Veterinary Pathology, College of Veterinary Medicine, Jeonbuk National University, Iksan, Republic of Korea

**Keywords:** bilateral parathyroid carcinoma, cat, parathyroid neoplasia, parathyroidectomy, primary hyperparathyroidism

## Abstract

Primary hyperparathyroidism (PHPTH) is a rare endocrine disorder in cats, and bilateral parathyroid carcinoma is exceptionally uncommon. A 14-year-old neutered male Russian Blue cat presented with decreased appetite, reduced activity, vomiting, and persistent hypercalcemia. Hypercalcemia was documented by increased ionized calcium (iCa; 1.90 mmol/L) and total calcium (14.3 mg/dL), and an increased intact parathyroid hormone concentration (18.6 pg/mL) supported a diagnosis of PHPTH. Cervical ultrasonography, computed tomography, and fine-needle aspiration cytology identified bilateral parathyroid-associated lesions but could not determine malignancy or the functional contribution of each lesion. During preoperative stabilization with intravenous 0.9% sodium chloride and furosemide, the iCa concentration decreased from 1.90 to 1.47 mmol/L. On the fourth day of hospitalization, complete right thyroid lobectomy with excision of the associated right parathyroid mass and left parathyroidectomy with partial left thyroidectomy were performed. Histopathologic examination supported the diagnosis of synchronous bilateral low-grade, well-differentiated parathyroid carcinomas, with infiltrative growth into adjacent non-neoplastic parathyroid tissue in the left lesion and focal capsular penetration in the right lesion. Postoperative hypocalcemia was managed with intravenous calcium gluconate and oral calcitriol during hospitalization, followed by oral vitamin D supplementation after discharge. Serum iCa normalized by postoperative day 28 and remained within the reference interval thereafter. On postoperative day 161, serum iCa and iPTH concentrations were within their respective reference intervals, and follow-up CT showed no evidence of local recurrence or detectable metastasis. This case highlights that bilateral parathyroid carcinomas may exhibit subtle histopathologic evidence of malignancy that is not predicted by preoperative imaging or cytology, while lesion-specific functional status and anatomical relationships to the thyroid gland may independently influence surgical planning.

## 1 Introduction

Primary hyperparathyroidism (PHPTH) is rare in cats and is most commonly caused by functional parathyroid adenomas (1–4), whereas parathyroid carcinoma is rare and poorly characterized (5, 6). Feline PHPTH typically presents with persistent hypercalcemia and nonspecific clinical signs, and most reported cases involve solitary, unilateral lesions; bilateral or multifocal disease is uncommon (1, 3, 4).

Preoperative imaging and cytology may not reliably distinguish benign from malignant parathyroid lesions (4, 7), and histopathologic distinction can be challenging in equivocal cases (8, 9). In cases with bilateral or multifocal parathyroid lesions, determining the functional contribution of each lesion presents an additional challenge. PTH measurement in fine-needle aspiration (FNA) washout fluid can help identify parathyroid tissue and provide lesion-specific information on PTH secretion (6, 10). However, surgical planning also depends on each lesion’s anatomical relationship to the thyroid gland and surrounding structures, which may influence the feasible extent of resection.

This report describes a cat with primary hyperparathyroidism associated with synchronous bilateral parathyroid carcinomas. Diagnostic evaluation and surgical planning were complicated by uncertainty regarding the functional contribution of each lesion and their differing anatomical relationships to the thyroid gland. Histopathologic classification was also challenging because both tumors were well differentiated and mitotically inactive, with malignancy supported by subtle but distinct histomorphologic features.

## 2 Case description

A 14-year-old neutered male Russian Blue cat (5.32 kg) was presented with a 10-day history of decreased appetite and activity, with vomiting occurring once or twice daily. Diarrhea was not observed. The presence of polyuria and polydipsia could not be reliably assessed based on the owner’s observations; however, the urine specific gravity (USG) was 1.014 at presentation. Rectal temperature was 38.5 °C, heart rate was 160 beats/min, respiratory rate was 66 breaths/min, and systolic blood pressure was 140 mmHg.

A complete blood count (CBC), performed using a ProCyte Dx hematology analyzer (IDEXX Laboratories, Inc., Westbrook, ME, USA), was unremarkable. Routine serum biochemical analyses were performed using a PT10V veterinary chemistry analyzer (Samsung Electronics Co., Ltd., Suwon, Republic of Korea). Serum symmetric dimethylarginine (SDMA), aspartate aminotransferase, and total thyroxine were measured using a Catalyst Dx chemistry analyzer (IDEXX Laboratories, Inc., Westbrook, ME, USA), and ionized calcium was measured using a GEM Premier 5000 blood gas analyzer (Instrumentation Laboratory, Bedford, MA, USA). Serum biochemistry revealed azotemia (creatinine, 3.2 mg/dL; reference interval [RI], 0.7–2.1 mg/dL), hypercalcemia (ionized calcium [iCa], 1.90 mmol/L; RI, 1.11–1.46 mmol/L; total calcium [tCa], 14.3 mg/dL; RI, 8.9–12.6 mg/dL), and hypophosphatemia (phosphorus, 2.2 mg/dL; RI, 2.6–6.4 mg/dL). Serum SDMA was increased at 17 μg/dL (RI, 0–14 μg/dL). The complete serum biochemical results obtained at initial presentation are provided in Supplementary Table 1. Differential diagnoses for hypercalcemia included PHPTH, idiopathic hypercalcemia, neoplasia, vitamin D toxicosis, and chronic kidney disease. Concurrent renal disease was considered particularly because of the azotemia, USG of 1.014, and increased serum SDMA concentration.

On reevaluation, persistent hypercalcemia (iCa, 1.90 mmol/L; RI, 1.11–1.46 mmol/L) was present concurrently with an elevated intact parathyroid hormone (iPTH) concentration (18.6 pg/mL; RI, 2.0–14.0 pg/mL). Serum 25-hydroxyvitamin D [25(OH)D] was 83.6 ng/mL (VDI Laboratory Services, Chatsworth, CA, USA), classified as insufficient according to the laboratory’s criteria (deficient: ≤40.0 ng/mL, insufficient: 40.1–99.9 ng/mL, and sufficient: 100.0–150.0 ng/mL). These findings supported a diagnosis of PHPTH, while the 25(OH)D concentration was not suggestive of vitamin D toxicosis. Diagnostic imaging was performed to localize and characterize the suspected parathyroid lesions.

Thoracic and abdominal radiographs and abdominal ultrasonography were unremarkable. Cervical ultrasonography revealed an oval cystic mass protruding from the left thyroid lobe, containing a well-defined hypoechoic nodule measuring 8.4 × 5.2 mm in the left parathyroid region (Figure 1A). No mineralization or ultrasonographic evidence of local invasion was observed.

**Figure 1:**
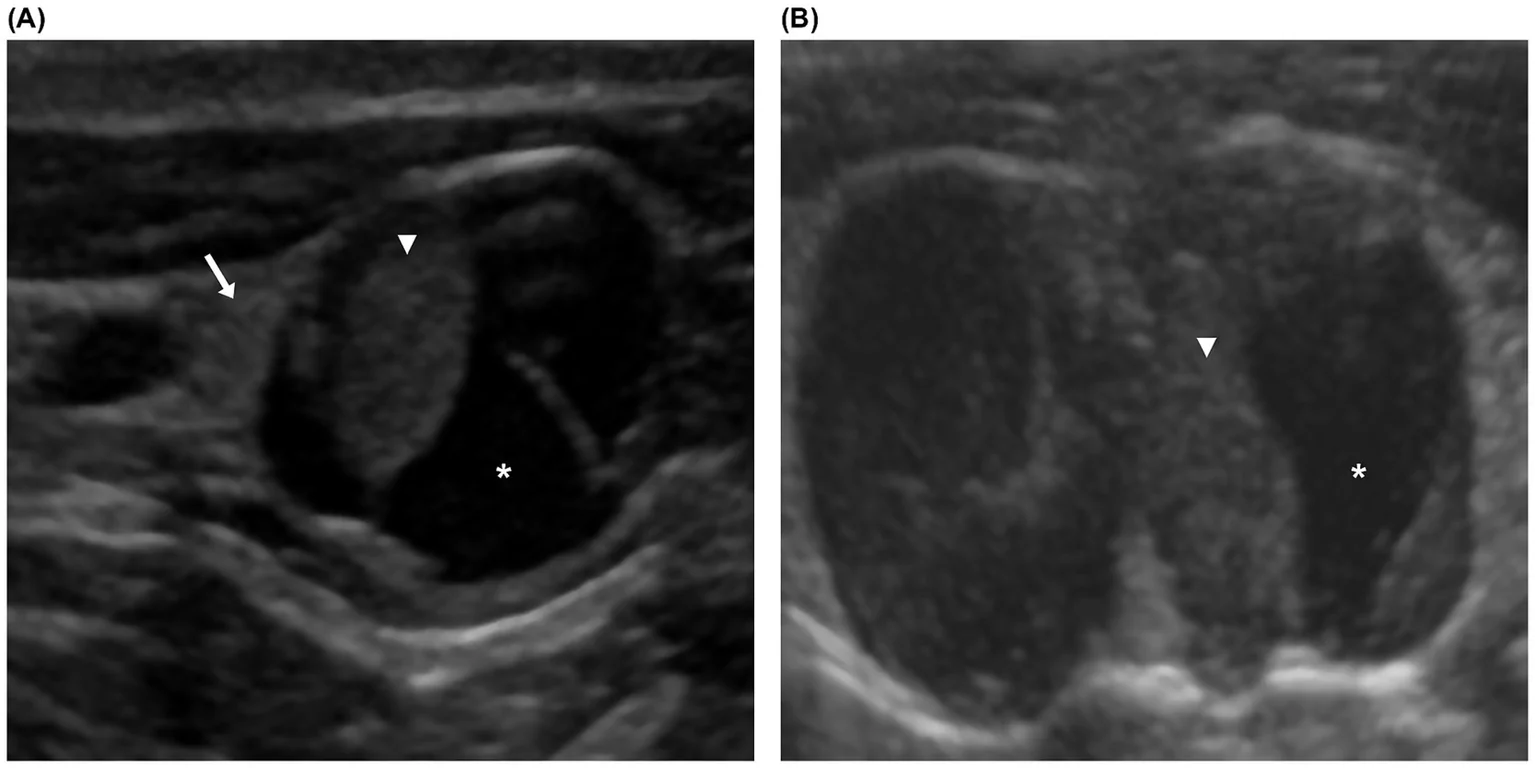
Ultrasonographic findings of bilateral parathyroid masses. **(A)** Transverse ultrasonographic image of the left parathyroid region showing an oval cystic mass (white asterisk) protruding from the left thyroid lobe (white arrow). A well-defined intracystic solid nodule measuring 8.4 × 5.2 mm is indicated by the white arrowhead. **(B)** Sagittal ultrasonographic image of the right parathyroid region showing a heterogeneous mass composed of solid (white arrowhead) and cystic (white asterisk) components.

Right cervical ultrasonography showed a well-circumscribed heterogeneous mass (10.0 × 13.0 mm) adjacent to the thyroid lobe, with moderately hypoechoic solid and anechoic cystic components (Figure 1B). Although its origin was uncertain, the findings were considered consistent with a parathyroid-associated lesion.

Ultrasound-guided FNA of both lesions showed round to polygonal cells singly or in loose aggregates, with fine chromatin and moderate anisokaryosis, suggestive of a well-differentiated endocrine neoplasm of probable parathyroid origin. Histopathology was recommended to assess malignancy.

Contrast-enhanced computed tomography (CT) was performed to assess lesion extent and anatomy for surgical planning (Figure 2). The left mass protruded from the caudal thyroid margin, measured 15.2 × 9.3 × 8.9 mm, and contained hypoattenuating soft tissue and central non-enhancing cystic components. The larger right mass (25.8 × 13.4 × 9.5 mm) was adjacent to the right thyroid lobe and had similar components. No regional lymphadenopathy or CT evidence of vascular or local invasion was identified. Surgery comprised complete right thyroid lobectomy with excision of the associated parathyroid mass and left parathyroidectomy with partial left thyroidectomy.

**Figure 2:**
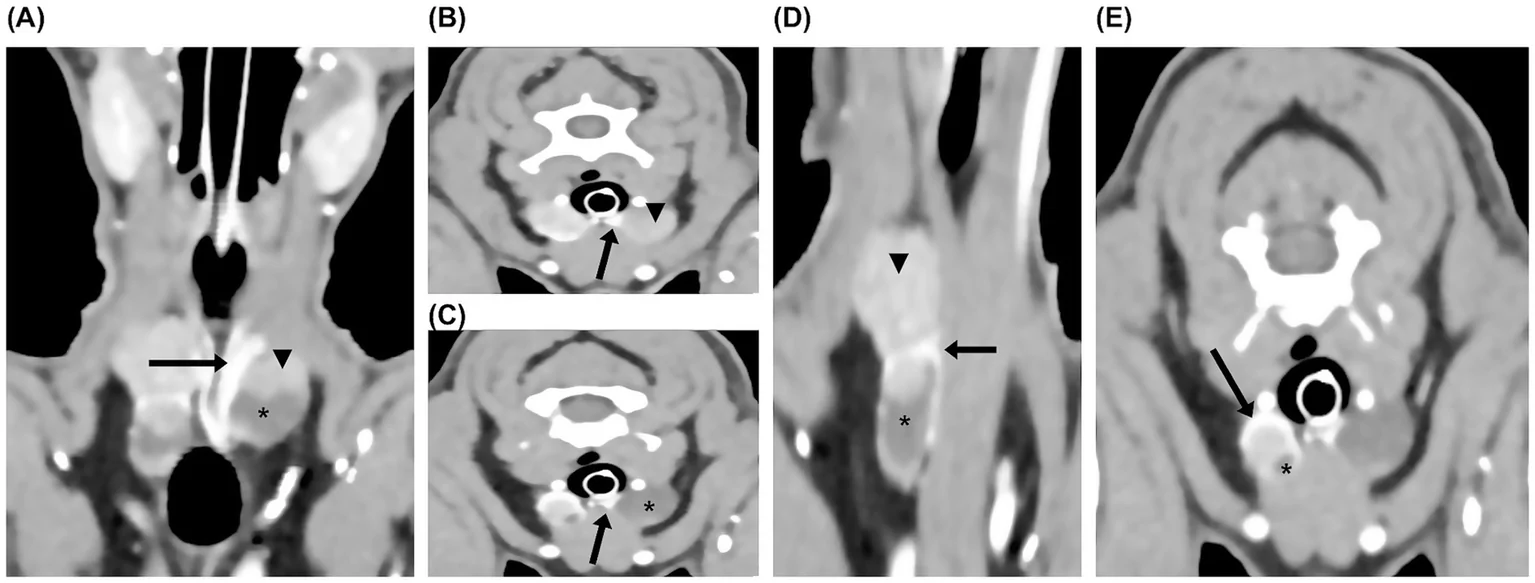
Computed tomographic (CT) findings of bilateral parathyroid masses. **(A–C)** Post-contrast dorsal **(A)** and transverse **(B, C)** CT images showing a left parathyroid mass composed of a hypoattenuating parenchymal component and a non-enhancing cystic component. The parenchymal component is indicated by black arrowheads **(A, B)**, the cystic component by black asterisks **(A, C)**, and the adjacent thyroid lobe by black arrows **(A–C)**. **(D, E)** Post-contrast sagittal **(D)** and transverse **(E)** CT images showing a right parathyroid mass composed of a hypoattenuating parenchymal component (black arrowhead in **D**) and a non-enhancing cystic component (black asterisks in **D** and **E**), with marked compression of the adjacent normal thyroid tissue (black arrows).

The cat was hospitalized for preoperative stabilization, with surgery on day 4. During days 1–2, two concurrent 0.9% sodium chloride-based intravenous (IV) infusions were administered at a combined rate of 4 mL/kg/h, one containing furosemide to deliver 1 mg/kg/h; an additional 2 mg/kg IV furosemide dose was given on day 1. On day 3, the furosemide infusion was discontinued, 0.9% sodium chloride was continued at 4 mL/kg/h, and furosemide was administered at 2 mg/kg IV twice daily. Serum iCa decreased from 1.90 to 1.47 mmol/L by surgery, while phosphorus increased from 2.4 mg/dL to 4.0 mg/dL.

Mild azotemia persisted during hospitalization, with creatinine concentrations ranging from 2.4 to 3.1 mg/dL. The transient increase in creatinine was considered potentially related to furosemide-associated volume contraction despite concurrent intravenous fluid therapy. Given the reduction in iCa concentration and the risks of persistent hypercalcemia, surgery proceeded as planned.

General anesthesia was induced with propofol following premedication with fentanyl, midazolam, and ketamine and maintained with sevoflurane in oxygen. Intraoperative analgesia was provided using continuous rate infusions of fentanyl and ketamine, and intravenous crystalloid fluids were administered throughout the procedure.

A ventral midline cervical approach was used to access the thyroid and parathyroid glands. The right thyroid lobe was completely excised with the associated parathyroid mass. The right-sided lesion was closely associated with and partially embedded within the thyroid parenchyma, making separation from the thyroid lobe impractical. On the left side, the parathyroid mass was completely excised with partial resection of the adjacent thyroid tissue while preserving the uninvolved portion of the left thyroid lobe (Figure 3). Hemostasis was achieved using bipolar electrocautery. No intraoperative complications occurred, and the surgical site was closed routinely in multiple layers.

**Figure 3:**
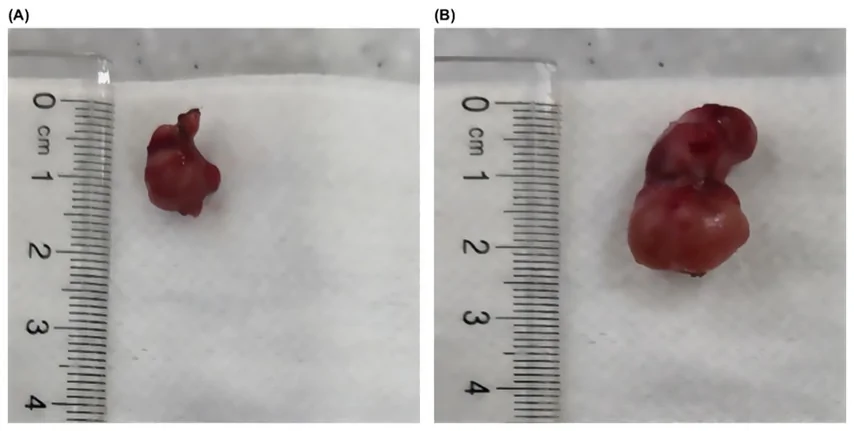
Gross surgical specimens of the resected thyroid and parathyroid tissues. **(A)** Excised left parathyroid mass with adjacent thyroid tissue following partial thyroidectomy. **(B)** Excised right thyroid lobe and associated parathyroid mass following complete right thyroid lobectomy.

The excised tissues were submitted for histopathologic examination. Histopathologic evaluation of the left parathyroid mass revealed a well-differentiated endocrine epithelial neoplasm arranged in solid sheets and coalescent nodules within a delicate fibrovascular stroma. The neoplastic cells exhibited a polyhedral morphology with moderate amounts of eosinophilic cytoplasm and round to oval nuclei displaying modest anisocytosis and anisokaryosis. No mitotic figures were observed, and there was no evidence of vascular or lymphatic invasion. However, the neoplastic population displayed areas of modest infiltrative growth, extending into adjacent non-neoplastic parathyroid gland tissue (Figure 4A) and the fibrous capsule. Additionally, perivascular palisading of neoplastic cells around small blood vessels was observed (Figure 4B, arrow), adjacent to basaloid parathyroid cells (Figure 4B, asterisk). Neoplastic tissue extended to the histologic margins. Although the neoplastic cells were overall well differentiated and lacked mitotic activity, the infiltrative growth supported a diagnosis of low-grade parathyroid carcinoma.

**Figure 4:**
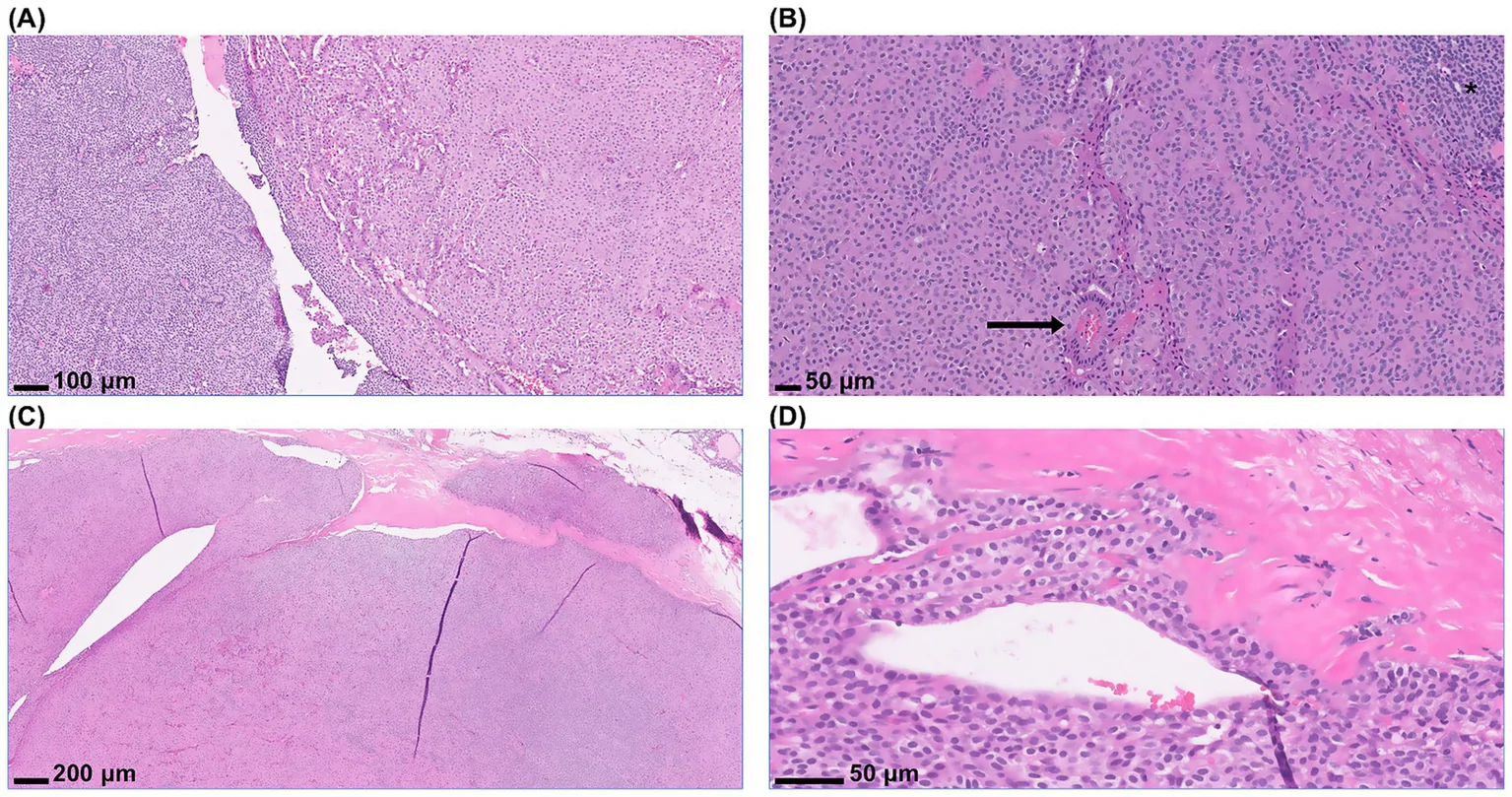
Histopathologic examination of the bilateral parathyroid tumors (hematoxylin and eosin stain, H&E). **(A)** Left parathyroid gland. Mild infiltration of eosinophilic neoplastic chief cells into areas of adjacent basaloid parathyroid cells (scale bar = 100 μm). **(B)** Left parathyroid gland. Perivascular palisading of neoplastic parathyroid cells around a small-caliber blood vessel (black arrow), adjacent to basaloid parathyroid cells (black asterisk) (scale bar = 50 μm). **(C, D)** Right parathyroid gland. Focal extension of compact, well-differentiated neoplastic parathyroid chief cells into the fibrous capsule, interpreted as capsular penetration, viewed at low (C, scale bar = 200 μm) and high (D, scale bar = 50 μm) magnification.

Microscopic evaluation of the mass associated with the right parathyroid gland, located within and immediately adjacent to the thyroid tissue, revealed a well-differentiated endocrine epithelial neoplasm arranged in cohesive nests, clusters, and solid sheets within a delicate fibrovascular stroma. The neoplastic cells were polyhedral, with moderate amounts of eosinophilic cytoplasm and relatively uniform round to oval nuclei showing mild anisocytosis and anisokaryosis. No mitotic figures were observed, and there was no evidence of vascular or lymphatic invasion. Focal areas of the neoplasm extended into the fibrous capsule, consistent with capsular penetration (Figures 4C,D). Although overt infiltrative invasion was not identified, the presence of focal capsular penetration in an otherwise well-differentiated parathyroid neoplasm was considered supportive of its interpretation as a low-grade parathyroid carcinoma. The mass was completely excised, with neoplastic tissue extending to within 1 mm of the specimen margin.

Postoperatively, iCa decreased to 1.27 mmol/L (RI, 1.11–1.46 mmol/L) and to 1.02 mmol/L within 24 h. On postoperative day 1, 10% calcium gluconate (0.6 mL/kg IV over 15 min) was administered twice and oral calcitriol (0.01 μg/kg twice daily) was initiated, increasing to 0.015 μg/kg twice daily on day 2. Calcitriol was continued through day 4 but discontinued after discharge because the owner could not administer it. Oral vitamin D supplementation was then continued using alternative preparations and adjusted based on serial calcium measurements. iCa normalized by day 28 (1.15 mmol/L) and remained within the reference interval on day 42 (1.26 mmol/L). Vitamin D supplementation was discontinued on day 70 after calcium concentrations stabilized.

Postoperative renal parameters were serially monitored. Creatinine decreased from 3.2 mg/dL preoperatively to 2.4 mg/dL on postoperative days 1–2 and remained at 1.6–2.2 mg/dL through day 161. Blood urea nitrogen was 16–31 mg/dL from day 3 onward, measured SDMA concentrations remained within the reference interval, and USG was 1.023–1.030 on available urinalyses. Total thyroxine (T4) remained within the reference interval on days 1, 7, 14, 70, and 161 (1.0, 1.0, 1.2, 1.6, and 0.8 μg/dL, respectively; RI, 0.8–4.7 μg/dL), although the day 161 value was at the lower limit of the reference interval. Serum thyroid-stimulating hormone (TSH) was not measured.

At the final follow-up on postoperative day 161, serum iCa (1.43 mmol/L) and iPTH (4.7 pg/mL) concentrations remained within their respective reference intervals, whereas serum 25(OH)D concentration was 69.8 ng/mL and was again in the insufficient range. Vitamin D supplementation was resumed at that visit. Follow-up CT on postoperative day 161 revealed no evidence of local recurrence or detectable metastasis. No further follow-up was available.

## 3 Discussion

Parathyroid tumors are rarely reported in cats, with most reported cases involving adenomas (11). Parathyroid carcinoma is exceedingly uncommon, and bilateral parathyroid neoplasia is exceptionally rare (6, 12). The present case documents synchronous bilateral parathyroid carcinomas and illustrates the diagnostic and surgical challenges associated with bilateral parathyroid neoplasia in cats.

In cats, parathyroid adenomas are typically functional, causing excessive PTH secretion and hypercalcemia (1–4). Although rare, parathyroid carcinomas may also be hormonally active and contribute to hypercalcemia (1, 3, 5, 6). In a study of 32 cats undergoing surgery for PHPTH, including seven with parathyroid carcinoma, postoperative normalization of serum calcium provided indirect evidence of functional activity (4). Markedly increased PTH concentrations in cystic fluid aspirated from a feline parathyroid carcinoma provided direct lesion-specific evidence of PTH secretion (6). Together, these findings support the functional potential of feline parathyroid carcinoma.

On preoperative imaging, both cervical lesions were well circumscribed with solid and cystic components. The right lesion was larger, but neither showed definitive imaging features of malignancy. FNA yielded similar endocrine-type cytologic findings from both lesions. Thus, imaging and cytology supported bilateral parathyroid-associated lesions but could not reliably distinguish benign from malignant disease preoperatively.

Histopathology, however, identified distinct features supporting malignancy in each lesion. Distinguishing benign from malignant parathyroid neoplasms can be difficult in well-differentiated tumors lacking overt cytologic atypia or mitotic activity (8, 9), making careful assessment for invasive or infiltrative growth important in equivocal cases (9). In cats, invasion into adjacent thyroid tissue has also been described in parathyroid carcinoma (6). In this case, both tumors were well differentiated and mitotically inactive, but infiltrative growth into adjacent non-neoplastic parathyroid tissue in the left lesion and focal capsular penetration in the right lesion were the principal histomorphologic criteria supporting their interpretation as low-grade parathyroid carcinomas.

Bilateral cystic parathyroid carcinoma has previously been reported in a cat (6); therefore, bilateral occurrence or functional activity alone is not the principal significance of this case. Rather, the present case documents synchronous bilateral, low-grade, well-differentiated parathyroid carcinomas with subtle but distinct histopathologic evidence of malignancy: infiltrative growth into adjacent non-neoplastic parathyroid tissue in the left lesion and focal capsular penetration in the right. Neither feature was predicted by preoperative imaging or cytology. PTH-FNA washout could have provided lesion-specific functional information for surgical planning but would not distinguish benign from malignant disease. The lesions also differed substantially in their anatomical relationships to the thyroid gland, requiring different surgical approaches. Thus, these findings illustrate complementary roles of histopathology, lesion-specific functional assessment, and surgical anatomy in bilateral parathyroid disease.

Lesion-specific functional contribution represents a separate consideration from malignancy. Although PTH-FNA washout does not distinguish benign from malignant parathyroid disease, it may inform surgical planning by defining each lesion’s functional contribution. This is particularly relevant in bilateral disease, where tumor resection must be balanced against preservation of residual parathyroid function. In this case, persistent preoperative hypercalcemia and postoperative hypocalcemia supported excess PTH secretion from at least one resected lesion, although the relative functional contribution of each lesion remained undetermined.

Complete right thyroid lobectomy with excision of the associated parathyroid mass was performed, whereas the left mass was excised with partial left thyroidectomy. Intrathyroidal or deeply thyroid-associated parathyroid masses may require ipsilateral thyroidectomy because intraparenchymal location and close vascular relationship can limit surgical separation (13, 14). The larger right lesion was partially embedded within the thyroid parenchyma, making partial resection anatomically impractical.

Preservation of uninvolved left thyroid tissue after complete right thyroid lobectomy was intended to minimize thyroid loss and reduce the risk of postoperative hypothyroidism (13, 15). Calcium homeostasis primarily depends on functional parathyroid tissue; extensive or bilateral parathyroid excision may cause transient or permanent hypoparathyroidism and subsequent hypocalcemia, particularly when residual functional tissue is limited or suppressed (13, 15). Postoperative hypocalcemia requiring calcium and calcitriol supplementation in this cat was consistent with this recognized consequence.

Gradual normalization of iCa over several weeks was consistent with the reported postoperative course after removal of hyperfunctioning parathyroid tissue (1, 4). Although lesion-specific functional status remained unknown, this course supported the presence of functional parathyroid tissue among the resected lesions and underscored the need for postoperative calcium monitoring and individualized supplementation. At approximately 5 months, the cat was clinically stable and normocalcemic, with no evidence of local recurrence or detectable metastasis on follow-up CT.

The postoperative calcium course differed from that reported by Virtanen et al. (6), in which extensive bilateral parathyroid and thyroid resection was not followed by clinically relevant hypocalcemia, whereas prolonged postoperative hypocalcemia occurred in this cat. Although direct comparison between individual cases is limited, these outcomes illustrate variability in postoperative calcium responses after bilateral parathyroid surgery, with contributing factors remaining uncertain.

Several limitations warrant consideration. First, preoperative PTH-FNA washout was not performed, precluding lesion-specific functional assessment. Given the established PHPTH and bilateral parathyroid-associated lesions on imaging, PTH-FNA washout might have clarified the relative functional contribution of each lesion and informed surgical planning (6, 10).

Second, an immunohistochemical (IHC) panel including parafibromin, galectin-3, and Ki-67 was not performed. Benign and malignant parathyroid neoplasms may overlap morphologically, particularly in well-differentiated tumors lacking overt cytologic atypia or mitotic activity (8, 9). In human endocrine pathology, loss of parafibromin expression, increased Ki-67 labeling, and galectin-3 positivity are adjunctive markers in equivocal cases (16), but species-specific validation and routine standardization in feline parathyroid neoplasia are lacking. IHC could therefore have provided additional immunophenotypic and proliferative information. Nevertheless, the diagnoses were based on routine H&E findings, principally infiltrative growth into adjacent non-neoplastic parathyroid tissue on the left and focal capsular penetration on the right.

Lastly, serum iPTH concentration was not measured at every postoperative visit, and follow-up beyond 5 months was unavailable. Postoperative response was monitored primarily by physical examination and serial total calcium and iCa measurements, documenting resolution of hypocalcemia and maintenance of iCa within the reference interval. At approximately 5 months, serum iPTH and calcium concentrations were within their reference intervals, and contrast-enhanced CT showed no evidence of local recurrence or detectable metastasis. The absence of serial iPTH measurements limited assessment of temporal parathyroid recovery and longitudinal biochemical surveillance, while short follow-up precluded evaluation of long-term disease control. Continued surveillance with periodic iPTH and calcium measurements is warranted (6).

## 4 Conclusion

This report describes a rare presentation of feline primary hyperparathyroidism associated with synchronous bilateral, low-grade, well-differentiated parathyroid carcinomas. The case highlights the diagnostic and surgical challenges associated with bilateral parathyroid neoplasia. Preoperative imaging and conventional cytology identified bilateral parathyroid-associated lesions but could not reliably establish malignancy or determine lesion-specific functional contribution. Histopathologic diagnosis was also challenging because both tumors were well differentiated and mitotically inactive, with malignancy supported by subtle but distinct histomorphologic features in each lesion. Lesion-specific functional assessment, such as PTH-FNA washout, may provide additional information for surgical planning; however, the anatomical relationship of each lesion to the thyroid gland may further influence the feasible extent of surgical resection. The principal contribution of this case is the demonstration that synchronous bilateral parathyroid carcinomas may be well differentiated and histologically subtle, with different features supporting malignancy in each lesion despite similar preoperative appearances. In bilateral disease, histopathologic assessment, lesion-specific functional evaluation, and the anatomical relationship of each lesion to the thyroid gland should therefore be considered as distinct but complementary factors in diagnosis and surgical planning.

## Data Availability

The raw data supporting the conclusions of this article will be made available by the authors, without undue reservation.
