# Supplementary material for: Primary hyperparathyroidism in a cat with synchronous bilateral parathyroid carcinomas: a case report
Source: Front Vet Sci. 2026 Oct 29;13:1930590. doi: 10.3389/fvets.2026.1930590 (PMC13756587; doi:10.3389/fvets.2026.1930590)
Supplement: Supplementary file 1 [file Table_1.DOCX]

**Supplementary Table 1. Serum biochemical and ionized calcium results obtained at initial presentation.**

| **Analyte** | **Result** | **Reference interval** | **Unit** |
| --- | --- | --- | --- |
| Blood urea nitrogen | 24.5 | 15–37 | mg/dL |
| Cholesterol | 86 | 50–230 | mg/dL |
| Total bilirubin | 0.1 | 0–1 | mg/dL |
| Total protein | 7.0 | 5.8–9.1 | g/dL |
| Total calcium | 14.3 H | 8.9–12.6 | mg/dL |
| Globulin | 3.9 | 2.6–5.1 | g/dL |
| Phosphorus | 2.2 L | 2.6–6.4 | mg/dL |
| Alkaline phosphatase | 51 | 9–109 | U/L |
| Amylase | 941 | 500–1,400 | U/L |
| γ-Glutamyl transferase | 1 | 0–2 | U/L |
| Creatinine | 3.2 H | 0.7–2.1 | mg/dL |
| Alanine aminotransferase | 71 | 13–109 | U/L |
| Glucose | 136 | 74–152 | mg/dL |
| Albumin | 3.1 | 2.2–4.1 | g/dL |
| Lipase | 26 | 0–32 | U/L |
| Symmetric dimethylarginine | 17 H | 0–14 | µg/dL |
| Aspartate aminotransferase | 70 H | 0–48 | U/L |
| Total thyroxine | 2.2 | 0.8–4.7 | µg/dL |
| Ionized calcium | 1.90 H | 1.11–1.46 | mmol/L |

**Note.** Routine serum biochemical analyses were performed using a PT10V veterinary chemistry analyzer (Samsung Electronics Co., Ltd., Suwon, Republic of Korea). Serum symmetric dimethylarginine, aspartate aminotransferase, and total thyroxine were measured using a Catalyst Dx chemistry analyzer (IDEXX Laboratories, Inc., Westbrook, ME, USA). Ionized calcium was measured using a GEM Premier 5000 blood gas analyzer (Instrumentation Laboratory, Bedford, MA, USA). H, above the reference interval; L, below the reference interval.
